# Nanomaterial-Based Sensing and Biosensing of Phenolic Compounds and Related Antioxidant Capacity in Food

**DOI:** 10.3390/s18020462

**Published:** 2018-02-04

**Authors:** Flavio Della Pelle, Dario Compagnone

**Affiliations:** Faculty of Bioscience and Technology for Food, Agriculture and Environment, University of Teramo, 64023 Teramo, Italy; fdellapelle@unite.it

**Keywords:** nanomaterials, polyphenols, food, antioxidant capacity, total polyphenols, sensors, biosensors, metal nanoparticles, nanostructured sensors

## Abstract

Polyphenolic compounds (PCs) have received exceptional attention at the end of the past millennium and as much at the beginning of the new one. Undoubtedly, these compounds in foodstuffs provide added value for their well-known health benefits, for their technological role and also marketing. Many efforts have been made to provide simple, effective and user friendly analytical methods for the determination and antioxidant capacity (AOC) evaluation of food polyphenols. In a parallel track, over the last twenty years, nanomaterials (NMs) have made their entry in the analytical chemistry domain; NMs have, in fact, opened new paths for the development of analytical methods with the common aim to improve analytical performance and sustainability, becoming new tools in quality assurance of food and beverages. The aim of this review is to provide information on the most recent developments of new NMs-based tools and strategies for total polyphenols (TP) determination and AOC evaluation in food. In this review optical, electrochemical and bioelectrochemical approaches have been reviewed. The use of nanoparticles, quantum dots, carbon nanomaterials and hybrid materials for the detection of polyphenols is the main subject of the works reported. However, particular attention has been paid to the success of the application in real samples, in addition to the NMs. In particular, the discussion has been focused on methods/devices presenting, in the opinion of the authors, clear advancement in the fields, in terms of simplicity, rapidity and usability. This review aims to demonstrate how the NM-based approaches represent valid alternatives to classical methods for polyphenols analysis, and are mature to be integrated for the rapid quality assessment of food quality in lab or directly in the field.

## 1. Introduction

Polyphenols are ubiquitous secondary metabolites present in plant foods [1]. The common structural feature of all polyphenols, the presence of phenolic hydroxyl group(s), is the basis of their in vitro and in vivo antioxidant activity. In general, reaction of an antioxidant with a free radical induces the loss of an electron, the molecule becomes oxidized and generates a weak, non-toxic free radical that is stable and unable to propagate the reaction. In food matrices, antioxidants have a broad action that include, for example, prevention of rancidity of fats, as well as decreasing the adverse effects of reactive species, such as reactive oxygen and nitrogen species (ROS and RNS) [2]. The term antioxidant has a multifaceted nature, however, from a general point of view, in foods, a suitable definition is that an antioxidant is a natural or synthetic substance added to products to prevent or delay their oxidative deterioration.

Antioxidants in foods can be natural (vitamin C, vitamin E, carotenoids, polyphenolic compounds such as flavonoids, phenolic acids, anthocyanins, proteins such as transferrin, ceruloplasmin or albumin, minerals as selenium) or synthetic compounds, such as butylated hydroxyanisole, butylated hydroxytoluene, gallates, etc. It is well recognized that vegetables, fruits, grains and beverages such as tea, juice and wine constitute significant sources of natural exogenous antioxidants [3].

Antioxidants can be also classified as primary (called also long-term) antioxidants and secondary (called also processing) antioxidants. The primary antioxidants behave mainly as radical captors, hydrogen donors or radical chain breakers, while secondary antioxidants are mainly known as peroxide decomposers or singlet oxygen quenchers [2]. 

Despite classical studies reported that the ‘protection’ action derives by the presence of antioxidants as vitamins C and E which are found in high amounts in fruits and vegetables, nowadays, PCs are assumed to play an equally important role in disease prevention [4].

### 1.1. Phenolic Componds and Health 

Oxidative stress is a destructive process caused by an increase in the production of ROS/RNS or a decrease in the antioxidant network that leads to an imbalance between the oxidative and antioxidant systems; this causes oxidative modification of biological macromolecules, tissue damage and can induce accelerated cellular death [5]. PCs including phenolic acids, flavonoids and proanthocyanins that are naturally widely distributed in plants as a protective mechanism against biotic and abiotic stresses. High intake of fruits, vegetables, grains, spices and herbs and other food sources of dietary polyphenols has been linked to lowered risk of most common degenerative and chronic disease [6]. Indeed, it has been suggested that an intake of a rich antioxidant diet is inversely associated with the risk of developing diseases linked to oxidative stress [3,6,7,8], and has been proved to have positive effect in the anti-inflammatory mechanisms [6,9,10].

Several recent reviews and experimental papers are devoted to the study of the effect of PCs, and definitely their antioxidant properties continue to attract the attention of the whole scientific community interested to health and food [3,6,7,8,9,10,11]. Epidemiological studies and associated meta-analyses strongly suggest that long term consumption of diets rich in plant polyphenols offers protection against the development of cancer, cardiovascular diseases, diabetes, osteoporosis and neurodegenerative diseases [3,6,7,8,9,10,11]. However, a direct in vivo antioxidant effect of polyphenols is questionable, because the concentrations in blood are low (nmols per liter) compared to other antioxidants; moreover, extensive metabolic reactions following ingestion decrease their antioxidant activity. This indicates that in vitro experiments with the original compounds found in food are not necessarily relevant to the in vivo situation. Thus, despite the existence of considerable data suggesting the benefits of (poly)phenol intake, conclusions regarding their potential role in prevention still remained unclear due to several limitations of existing studies. 

For this reasons, in recent years, the biological role of polyphenols and their influence on human health have been studied in depth with respect to their in vivo effects and their bioavailability [7,8,9,10,12,13].

Many studies have emphasized the importance of polyphenols because in many cases their activity is correlated to the AOC of a sample (food). Other studies have highlighted the importance of single molecules or groups of polyphenols present in specific foods. Indeed, dietary polyphenols present in different food matrices in many cases act as key modulators of signaling pathways and are therefore considered ideal chemopreventive agents. Dietary polyphenols also contribute to epigenetic changes associated with the fate of cancer cells and have emerged as potential drugs for therapeutic intervention. Polyphenols have also been shown to affect post-translational modifications and microRNA expression [6,7].

The consumption of extra virgin olive oil (EVOO), as example, a typical fat source of the Mediterranean diet, has been associated with a significant reduction of cancer risk. The EVOO PCs have been demonstrated to influence gene expression in the endocannabinoid system via epigenetic regulation in both in vitro and in vivo [14]. The action of resveratrol, a polyphenol found in red wine, on various pathologies (i.e., Alzheimer’s disease) has been certainly demonstrated [15]. Based on these considerations, several studies reported in the last few years were designed to validate sensitive and reliable translational tools to characterize brain bioavailable polyphenols. Different research groups worldwide with expertise in Alzheimer’s disease, plant biology, nutritional sciences, and botanical sciences have reported very high quality studies showing that polyphenols and their metabolites, originating from several dietary sources, including grapes, cocoa, etc., are capable of preventing Alzheimer’s disease and other neuro degenerative disorders [15]. It is then evident, today, the PCs protective impact on human health, and some of the possible mechanisms of action through which their metabolites may exert such effects are known.

### 1.2. Phenolic Compounds and Food Technology 

It should be noted that the activity of antioxidants in foods and biological systems depends on a multitude of factors, including the colloidal properties of the substrates, the conditions and the stages of oxidation and the localization of antioxidants in different phases. PCs, ubiquitous in plants, are of considerable interest and have received increasing attention in recent years because on the top of bioactivity they have technological functions influencing the final product processed as food. An idea of the different classes of PCs, their chemical structure and occurrence in different foods can be found looking at the database phenol-explorer.eu [16]. Generally, nowadays, foods manufactured from fruits and vegetables, that also contain polyphenols in significant amounts [1]; in this respect they are considered amongst the most desirable phytochemicals due to their unique properties [17].

PCs can play a major role in preventing or delaying autoxidation and have attracted much attention as food stabilizers, dietary supplements and natural health products. Polyphenol-rich foods are widely used in formulated products and an ever increasing number of research papers have appeared in the recent literature on the discovery and application of these natural antioxidants and their therapeutic and technological properties [1,18].

The use of foods rich in polyphenols, plant extracts and their derived products in various food and beverage formulations is an increasing trend in the food industry. Selection of these plant extracts and their application depends on the functional properties, availability, cost effectiveness, consumer awareness and their effect on the sensory attributes of the final product. For example green tea extract and grape seed extract are two popular plant extracts that have been widely used in various food and beverage formulations [19]. Polyphenols may contribute to the bitterness, astringency, color, flavor, odor and oxidative stability, as in the case of olive oil and chocolate. Antimicrobial activities against major food borne pathogens has been also recently reported [19]. These features make food phenols a potentially interesting material for the development of functional foods. 

### 1.3. Analysis of Phenolic Compounds and Antioxidant Capacity

In order to be used in specific formulations (food, pharmaceutical, cosmetic, etc.), as well as to be employed/evaluated for different research topics, PCs needs to be carefully analyzed and characterized [20]. The development of efficient procedures for the extraction, analysis, and characterization of PCs from different sources is a challenging task due to the structural diversity of PCs, the complex natural sources and their interaction with different cellular components [21]. Indeed, no unique analytical approach for the quantification of these compounds is used. In each particular case, the method that is most adequate to the particular tasks should be selected. An overview on the problems of chemical analysis of phenolic antioxidants has been given by Ziyatdinova and Budnikov in 2015 [21]. In this review natural phenolic properties and classification, together with selective and unselective methods were critically discussed.

Regardless of the analytical method used, in the majority of the cases an extraction step is required and it should be carefully optimized in order to achieve high accuracy. The sample preparation and extraction of PCs are carried out taking into account the nature of the investigation and chemical properties of phenolic antioxidants: molecular structure, polarity and concentration [21]. Furthermore, also the food matrix composition plays a critical role in this step (e.g., fatty matrices such as olive oil, chocolate; matrices rich in sugars or pigments, etc.). Thus, in the analysis of polyphenols no universal sample pretreatment method is used. 

The classical liquid-liquid extraction (LLE) gives generally a good recovery of PCs, however, it has some drawbacks, such as the requirement of a huge amount of solvents, long extraction time, limited choice of solvents, and, in some cases, the degradation of the target compounds. Other extraction techniques are represented by: solid-liquid extraction (SLE), solid phase extraction (SPE), pressurized liquid extraction (PLE), supercritical extraction (SCE), ultrasonic-assisted extraction (UAE), and microwave-assisted extraction (MAE) [20,21,22]. In recent years there has been a tendency towards the automation and the use of micro-extraction systems in the clean-up phase, with the aim of automating the clean-up procedure and reduction of solvent consumption, analysis times and matrix effects [23,24]. Certainly, the use of a sensor, or sensing system, ideally should require no, or minimal, sample pretreatment. Thus, this specific issue will not be covered in this review. 

With the advancement of analytical sciences, there has been an array of modern tools exploitable for polyphenols analysis, starting from high performance liquid chromatography (HPLC), gas chromatography (GC), liquid chromatography-mass spectrometry (LC-MS), gas chromatography-mass spectrometry (GC-MS), Fourier Transform infrared spectroscopy (FT-IR), nuclear magnetic resonance (NMR), capillary electrophoresis (CE), sensors and biosensors, among others [20,21,22]. The quantification of PCs using chromatography is the most frequently used approach since it is possible to identify and quantify selected compounds within complex matrixes. HPLC is very useful since compounds are separated because of their intrinsic chemical properties, such as hydrophobicity of aglycones and hydrophilicity of corresponding glycosides [25]. Reverse phase separation is classically the most used technique for separation of PCs [26], coupled to ultraviolet/visible, photodiode array, and UV-fluorescence detection. More recently, mass spectrometric (MS) detection has definitely taken over, being able to identify and quantify PCs at low levels in very complex mixtures [27]. These techniques are ideal for the identification of a single compound or a metabolite, however they require expensive equipment, trained personnel and may be time consuming. Moreover, separation techniques do not give information on the AOC of the polyphenols studied. Ignat et al. [22] reviewed in 2011 the analysis of PCs, from their extraction to their detection. Natural sources of polyphenols have been reported, and the review focused on the isolation and characterization techniques (in particular separative), with the related advantages as well as the limitations of each method.

The total amount of polyphenols and the evaluation of AOC related to PCs, both in the research and industries, it is often carried out with rapid and user-friendly screening assays. In spite of the great number of the methods proposed, there is no approved, standardized method, which can by itself provide an adequate measure of TP or AOC, because of the complexity of this issue. The Folin-Ciocalteu (FC) method has gained popularity to determine TP and it is commonly known as the TP (or phenolic) assay. However, the FC assay measures a sample’s reducing capacity, but this does not necessarily reflect the total amount of phenols. FC is based on the concept that Mo (VI) (yellow color in the complex Na_2_MoO_4_·2H_2_O) can be reduced to Mo (V) (the blue complex PMoW_11_O_4_)^4−^. The redox reaction occurs between Mo (VI) and the reductants (antioxidants). The FC assay is generally accepted as a method to determine TP and a huge volume of data based on this assay are available. It has been reported that this method overestimates the phenolic content to a high extent [28,29] and it is affected by the presence of various interferents (particularly reducing sugars and proteins commonly found in food) [28,30]. Nevertheless, the FC assay is one of the most used and important approaches. For this reason several works over the years have aimed to improve its performance. Sánchez-Rangel et al. [30], for example, described different methodologies to improve the FC assay specificity and usability, and the resulting advantages and disadvantages.

There are different possible classifications of methods to evaluate AOC. According to the type of oxidant agent used, scavenging capacity assays against specific ROS/RNS, or scavenging capacity assays against stable chemical radicals, can be found. Depending on the methodology employed, AOC assays can be classified as competitive or non-competitive. In competitive assays, the target species (called probe) is a compound that competes with antioxidant compounds for the reactive species. In non-competitive assays, antioxidant compounds interact with reactive species with no other competing target molecules in solution. Moreover, on the basis of the chemical reactions involved, the major AOC assays can be roughly divided into two categories: hydrogen atom transfer reaction-based assays (that monitor competitive reaction kinetics) and single electron transfer reaction-based assays (they involve a redox reaction with the oxidant). The most commonly used assays are based on spectrophotometric methods, such as scavenging activity toward ‘stable’ free radicals (DPPH• and ABTS• assays), reduction of metal ions (FRAP and CUPRAC assays) and competitive methods (ORAC and TRAP) [31]. The results obtained with these assays, in food analysis, are classically expressed as Trolox equivalent antioxidant capacity (TEAC). The principal assays for in vitro and in vivo antioxidant evaluation have been reviewed and classified in 2013 by Alam et al. [32]. The Alam et al. review aimed to list methods that are used to evaluate the antioxidant property of various samples, to reduce time for literature review and method development [32]. The work by López-Alarcón et al. [31] reported an overview of the state of art and classified the mechanisms of action of the ‘screening’ AOC assays. In this review the classical and non-classical mechanisms of action of antioxidant-containing natural products were also discussed.

Electrochemical (EC) techniques, by their very nature, can give direct determinations of the total AOC without using reactive species. These methods try to establish a relationship between oxidation potential and antioxidant activities using cyclic voltammetry (CV), differential pulse voltammetry (DPV), square wave voltammetry (SWV), flow-injection analysis with amperometric detection, and, more recently, microfluidic chip strategies coupled with amperometric detection [2,33]. The EC methods can be coupled to enzymes for the development of enzyme electrodes that allow the successful determination of the total AOC or TP evaluation in various media: foodstuffs and beverages, biological fluids, pharmaceuticals, etc. Recently, electrochemical approaches applied to the analysis of polyphenols have been reviewed in 2015 by Ziyatdinova and Budnikov [21], with particular attention to drugs and foods.

### 1.4. Nanomaterials

Nanomaterials (NMs) have received great attention in recent years in different fields due to their enormous potential, thanks to their excellent unique optical, electrical, magnetic, catalytic, biological, or mechanical properties. Nanomaterials are defined by the European Commission Communication of 7 June 2005 as [34]: ‘means and natural, incidental or manufactured materials containing particles, in an unbound state or as an aggregate or as an agglomerate and where, for 50% or more of the particles in the number size distribution, one or more external dimensions is in the size range 1 nm–100 nm’.

Methods of chemical analyses and sensing that take advantage of the unique properties of NMs, as well as new methods for the analysis of NMs themselves, are intense research areas. Nanomaterials have triggered the development of both new ways of performing target concentration and detection, and new analytical methods and instrumentation for measuring the properties of NMs sensitive to changes at the nanometer or single molecule level. In brief, NMs have special thermal, mechanical, optical, electrical, magnetic and biological properties, which are size- dependent and can be tuned by simply adjusting the size, the shape and the extent of agglomeration. The development of novel sensors and biosensors with interest for food industry and research is one of the key fields for nanobiotechnology and nanomaterial science.

Several new analytical approaches have been attempted in recent years to evaluate AOC and TP content in food samples. In the last decades, particular interest has been devoted to methods having advantages of sensitivity, rapidity, that require simple and relatively inexpensive instrumentation, and small sample volumes, with a general aim to rationalize the use of research resources. In this respect NMs have been employed in several polyphenols sensors and sensing strategies. Different scientific papers and reviews [35,36,37,38,39,40,41] describe the use of this nano-based approach underlying the advantages and the improvements of these materials. NMs and functionalized NMs are used as catalytic tools, immobilization platforms or as optical or electroactive labels to improve the biosensing performance exhibiting higher sensitivity, stability, and selectivity [37].

The aim of this review is to provide an updated critical overview of different recently reported nanomaterial-based sensing and biosensing strategies, based on various analytical approaches, for the evaluation of TP content in foods and AOC related to polyphenols. No further information on other separative techniques will be given. For each of the topics in the following paragraphs, the state of the art will be discussed, taking into account both published reviews and an updated selection of significant papers in the field. 

## 2. Optical Sensing 

Some noble metal nanoparticles (MNPs), beyond the already mentioned proprieties of NMs, exhibit a unique feature that allows their use in colorimetric or spectrophotometric sensing. In fact, under the influence of electromagnetic radiation, in the visible range, the electrons of the surface atoms can easily move through vacant orbitals generating absorption at a particular wavelength. This phenomenon is called localized surface plasmon resonance (LSPR). The coherent oscillations of the electrons in resonance with the frequency of the light give rise to LSPR [42], which can be explored in a wide range of applications in chemistry, biology and nanotechnology. 

Spherical MNPs can be generated by different kind of synthesis to obtain MNPs suspensions with different size and stability in different reaction ‘media’. The size and the distribution of the MNPs formed can vary significantly depending on the reaction conditions, i.e., the mild reducing agent employed, pH and chemical stabilizer used (generally called capping agent). A soluble salt containing the metal ion is very often the metal source. Under appropriate conditions, a colloidal dispersion can be obtained. It should be emphasized that both reduction and stabilization play a fundamental role in order to obtain a stable colloid. Antioxidant compounds, and particularly polyphenols, in appropriate optimized condition, are able to form MNPs, and, thus, can be studied via MNPs generation. 

**Figure 1:**
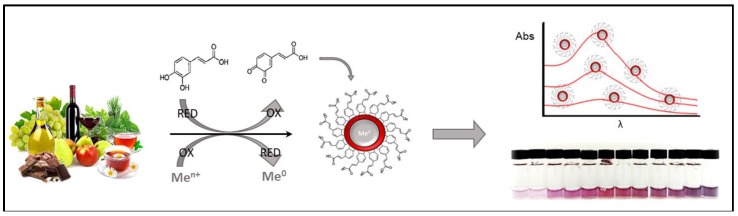
Schematic representation of AuNPs formation mediated by polyphenols. Colorimetric formation curve (caffeic acid) obtained following the Della Pelle et al. [46] strategy.

Vasilescu et al. in 2012 [43] reviewed the use of nanoparticles (NPs) and nanostructures for the development of analytical assays for the detection of food antioxidants; they focused both on optical and electrochemical strategies. The assays either exploit the ability of antioxidants to reduce noble metals leading to the formation of the corresponding nanoparticle or use antioxidants as reducing agents in the so called “seed-mediated” growth technique. In the latter approach, small NP seeds serve as nucleation centers for the growth and further increase in size of the nanoparticles. Since the absorbance of the colloidal NPs suspensions depends on the properties of the surface resonance plasmon bands, intense colorimetric and chromatic transitions are observed which are used as an index of antioxidant activity [44]. Optical strategies based on MNPs, the majority of which are based on AuNPs, have been treated in the review. Vilela et al. [45] also reviewed the subject in 2015, focusing on optical nanoparticle sensing in food and biological samples. The innovative use of MNPs (mostly gold and silver) and quantum dots (QDs) as novel tools for reliable assessment of antioxidant activity was reported. The authors defined these emerging nanoparticle-based optical strategies as novel, simple and inexpensive tools in the food and clinical rapid assay field. More recently, different strategies and NPs type have been proposed to develop novel NP-based ‘assays’ for antioxidant detection in food samples. Figure 1 schematically displays a generic formation of gold nanoparticles (AuNPs) mediated by food polyphenols and the resulting analytical response. Table 1 gives a comparison between the different NPs/QDS-based optical assay methods for polyphenol AOCs reviewed in this section.

Looking at the papers, some general aspects that makes a comparison difficult need to be emphasized. The difference in the detection of total TP and AOC detection is not always defined. Furthermore, the analytical performance (particularly in terms of sensitivity) of the reported methods are referred to different phenolic standards, that exhibit extremely different AOC (i.e., flavonols vs. monophenolic acids). Sample preparation is very often different. This can generate misunderstandings during any comparison of the performance of different methods based on totally different strategies. For this reasons we recommend the interested reader not to carry a simple comparison based on numeric data but rather to have a critical view of the methodology. The basis of the different approaches and the main outcomes have been reported in this section.

### 2.1. Gold Nanoparticles

Gold nanoparticles (AuNPs) have been the subject of the majority of the studies on MNPs; several works exploit the ‘catalytic properties’ and the large surface area of AuNPs for the quantitative analysis of PCs.

In the paper of Vilela et al. [47], the ability of endogenous food polyphenols to reduce Au(III) to AuNPs(0) in aqueous solvents was demonstrated and the AuNPs optical absorption peak due to the LSPR detected. The authors associated the AuNPs formation driven by different amounts of polyphenols to a sigmoidal function. The slope of the sigmoidal curve obtained was used as AOC index. The samples tested (four different plant extracts) were classified according to the increasing slopes. A good correlation between the slopes and the FRAP and FC methods (R = 0.900 and 0.970) was found while no significant correlation with ABTS• was observed (R = −0.730). This behavior was explained with the ability of the developed method to assess reducing power since FRAP and FC reflect the reducing power of the polyphenols in the sample. In addition, excellent correlation between the slope and TP obtained by HPLC-DAD was obtained (R = 0.930, *p*-value < 0.05). However, the slope obtained by the sigmoidal curve, give just a general indication of the AOC, since no polyphenol was used as standard to express the AOC as a numeric value. Using a similar analytical strategy Della Pelle et al. [46] synthesized AuNPs through a water-based mild chemical route (45 °C, 10 min) directly in a 0.5 mL volume. The AuNP formation was obtained using extra virgin olive oil (EVOO) endogenous polyphenols; also in this case the reaction was described by a sigmoidal curve. This new AuNPs-based AOC index was expressed in gallic acid equivalent antioxidant capacity units (GEAC) using gallic acid as reference polyphenol as for the majority of the AOC assays. The ratio of the slope of the sigmoid and concentration at half value of the maximun absorbance was found to be the optimal parameter to report the AOC. The data reported demonstrated that the compounds with *ortho*-diphenol (*o*-diphenol) functionality are the most active in reducing Au(III) to Au(0). A significant correlation among classical methods used to determine antioxidant activity (ABTS•, DPPH• and FC) and the proposed method was found with R values in the 0.960–0.970 range. The developed method requires, however, in the case of fat rich samples, extraction of polyphenols as in any other classical method. This drawback was overcome driving the AuNPs synthesis with an extraction-free approach using dimethyl sulfoxide (DMSO) as organic solvent [48]. DMSO solubilized the sample and stabilized the AuNPs suspension working as well as a cryogenic protector avoiding solidification at the temperatures used to block the synthesis. AuNPs formation was achieved in 5 min at 45 °C. Calibration curves were obtained for the typical polyphenols of EVOO (hydroxytyrosol, oleuropein and tyrosol) and chocolate (quercetin, caffeic acid and epicatechin) by simply reading the absorbance at 540 nm at the maximum LSPR. The method was applied to the determination of TPs in olive oil and chocolate samples. An excellent correlation for olive oil samples (R = 0.990, *n* = 28) and for chocolate samples (R = 0.905, *n* = 16) was achieved. 

Tułodziecka et al. [49] proposed a method to assess AOC of *Brassica* oilseeds (rapeseed), white flakes and meal extracts. The pH of the reaction mixture, incubation time and temperature, as well as the conditions of sample preparation (solvent polarity) were carefully optimized, studying the reactivity of sinapic acid, caffeic acid, gallic acid, ferulic acid and quercetin. The assay was based on the formation of gold AuNPs in an acetic buffer medium (pH = 4.6); after 60 min the absorbance of purple solutions left in the dark at room temperature was measured at 540 nm. The relationships between the AOC of rapeseed, white flakes and meal extracts analyzed by the AuNPs assay was compared with FRAP, DPPH• and FC methods. Positive significant correlations (R = 0.840–0.970) were found. In all cases the sample requires an extraction step. Interestingly, principal component analysis (PCA) of the data revealed the influence of processing operations and samples preparation on the AOC assays determined by different analytical methods, particularly in the AuNPs-based protocol. 

In the last years paper-based devices [63,64] have been proposed as simple and low cost tools to run simple in-field assays, Nery et al. [63] have reported an overview on this strategy. To this end Choleva et al. [44] have proposed a paper-based device in the form of a sensor patch that enables the determination of antioxidant activity through analyte-driven formation of AuNPs. Nucleation of gold ions on-paper to form NPs was exploited upon reduction by antioxidant compounds present in aqueous samples. The chromatic transitions, induced on the paper surface, were used to assess the antioxidant strength of the solution. When tested vs. different antioxidant molecules, intensity of the color response was observed in this order: catechin, gallic acid > caffeic acid > ascorbic acid > coumaric acid > vanillic acid > ferulic acid > cinnamic acid. This is in agreement with the antioxidant polyphenols classical reactivity (i.e., flavonoids > *o*-phenol > *m*-phenols) [41,43,60]. Antioxidant activity of tea and wine samples was assessed expressing the data as gallic acid equivalents and using visual inspection by a digital camera. Noticeably, the paper-based sensors stored under moisture-free and low temperature conditions do not lose their activity for several months. 

### 2.2. Silver Nanoparticles

The total number of papers based on the formation of silver nanoparticles (AgNPs) for the study of polyphenols and their reducing power is very low compared with those on AuNP-based assays. The synthesis of AgNPs using different reducing compounds and natural extracts has been reported, because of the great interest into the “green” synthesis of AgNPs [65] used in nanomedicine and microbiological applications [66,67]. The standard redox potential of the Ag/Ag^+^ couple suggest a higher reducing power with respect to the most commonly used Au/Au^3+^ pair. However, the polyphenol-mediated AgNPs formation occurs with extremely fast kinetics causing aggregation, collapse of the NPs or unstable NPs suspensions. 

A colorimetric method was proposed by Özyürek [50] based on the citrate-stabilized silver ‘seed growth’ mediated by polyphenols. After the AgNPs seed generation with citrate, reduction of Ag^+^ to AgNPs at 25 °C for 30 min by polyphenols produces an increase of the LSPR with peak maximum at 423 nm. TEAC values of various antioxidants (15 polyphenols including flavonoids, simple phenolic acids and hydroxycinnamic acids) using this method were comparable to those of the CUPRAC assay. The developed method was used to screen the AOC of some commercial fruit juices and herbal teas; oxalate, citrate, fruit acids, amino acids, and reducing sugars were studied as possible interferents. The polyphenols standards were successfully classified according with their AOC and the interferents studied didn’t affect the assay.

Recently, Teerasong et al. [51] proposed a method based on poly(vinyl alcohol)-embedded silver nanoparticles (PVA-AgNPs), also based on seed mediated growth. Ag^+^ was reduced, by polyphenols, to Ag° and accumulated on the PVA-AgNP surface, leading to an increase in the size of particles. The PVA-AgNPs seeds were produced using an aqueous solution of PVA and AgNO_3_ under continuous stirring and heating (~90 °C). Hydroxyl groups formed on the polymer upon heating served as reducing units. The assay was tested/optimized using only gallic acid as polyphenol standard. Ginger samples were assayed by dissolving them in water at 80 °C and adding the PVA-AgNPs. The increase in absorbance of PVA-AgNP suspension (with a slight red shift) was related to AOC. The method was compared with the ABTS• assay, that gave higher but well correlated AOC values (R = 0.992).

Direct formation of AgNPs for the evaluation of AOC of rapeseed and its products was proposed for the first time by Szydłowska-Czerniak et al. [52]. In this work, a careful optimization of the sample preparation was carried out, studying the effect of different solvents and their influence on the method for rapeseed at different stages of processing (ground, flakes, press cake and meal samples). The AgNPs generation was carried out in an ammonium buffer medium (pH = 8.4) at room temperature and the absorbance of the yellow-orange suspension was measured after 60 min at 405 nm. Antioxidant capacity was expressed as micromoles of sinapic acid equivalents (SA) per gram of sample; sinapic acid is the predominant free phenolic acid in rapeseed. Good correlation (R = 0.756–0.851) among the proposed AgNPs and the FRAP, DPPH• and FC methods for all rapeseed extract were found. Also in this case PCA evaluation demonstrates differences among the total amounts of antioxidants in rapeseed samples extracted by different solvents.

### 2.3. Other Metal Nanoparticles 

In recent years other NPs were employed for the colorimetric evaluation of PCs in food. The ceria redox couple Ce(IV)/Ce(III) was employed by Sharpe et al. [53] for the realization of a nanoceria-based patch for the optical assay of antioxidants. The latter was based on the use of immobilized formed ceria NPs on filter paper. Spectroscopic studies indicate that different antioxidants (trolox, epigallocatechin gallate, L-ascorbic acid, gallic acid, vanillic acid, quercetin, caffeic acid) produce a visible colour change, or formation of charge transfer complexes, for high-concentration dispersions of ceria NPs. An indirect colour inhibition based method to assess AOC through inactivation of ceria NPs surface adsorbed superoxides (previously pretreated) is reported for a paper-based device. The colorimetric response was found proportional at the AOC and concentration of the antioxidant standards. AOC of teas and medicinal mushrooms are reported as GEAC; the authors claim a reactivity similar to the ORAC assay. 

The AOC of the rapeseed and its by-product extracts was also studied using a cerium oxide nanoparticle-based (CeONPs) assay [54]. The rapeseed antioxidants reduced cerium(IV) ions to red-purple solutions of cerium oxide nanoparticles, with a typical absorption band at 510 nm. The CeONPs method can be effectively used at pH 5.6 (acetate buffer) reading the absorbance after 30 min of incubation at room temperature (20 °C). The resulting CeONPs appear elliptical and rod-shaped. The AOC analyzed by the CeONPs assay and the results for all the studied extracts obtained by a previously described AgNPs assay [52] were expressed as sinapic acid equivalents and correlated significantly (R = 0.818, *p* = 0.007). However, the CeONPs method is less sensitive, compared with the AgNPs assay, and no significant correlation with classical methods was obtained. 

AOC evaluation of rapeseed oils at various stages of technological processes has been also reported by Szydłowska-Czerniak et al. [55] using iron oxide nanoparticles (IONPs). Ferric ions were reduced by oil extracts in acidic medium, with a resulting formation of yellow solutions of iron oxide NPs. After 50 min from the extracts addition, the absorbance of IONPs yellow-orange solutions was measured at 396 nm. The formed IONPs appear spherical in shape, fairly monodisperse and homogeneous and Fourier transform infrared spectroscopy (FTIR) data supported the presence of sinapic acid (used as standard) adsorbed on the IONPs surface. Antioxidant capacities determined by the IONPs-based method for acetonic and methanolic extracts of rapeseed oils significantly correlated with FRAP and DPPH• values (R = 0.979–0.985 and 0.917–0.951, *p* < 0.05, respectively).

An assay for the determination of PCs based on their interaction with citrate-capped rhodium nanoparticles (RhNPs) was proposed by Gatselou et al. [56]. PCs (catechins, gallates, cinnamates, and dihydroxybenzoic acids) change the size and, then, LSPR of RhNPs, giving rise to analyte-specific spectral and colour transitions in the rhodium NPs suspensions. The citrate-RhNPs had dark green-brown colour and exhibited strong broad absorption bands in the ultraviolet region near 200 nm and a continuous absorption pattern in the visible range. New absorbance peaks at 350 nm and 450 nm and 580 nm were observed. On the basis of these findings, two RhNPs-based assays that enable to obtain a fingerprint of the total phenolic content and total catechin content of tea samples have been developed and applied in tea samples. The results correlated with the commonly used methods for the TP (R = 0.927) and for catechin content quantification (R = 0.832). This reaction features allows a semi-qualitative assessment of phenols in tea samples. 

Sharpe et al. [57] developed a sensing array based on different MNP oxides (cerium oxide, titanyl oxalate, iron oxide, and zinc oxide) as a colorimetric method, on paper, for polyphenol detection/evaluation. The antioxidant detection mechanism was based on the ability of polyphenols to form surface complexes of characteristic colors with metal oxides, with unique spectral properties and characteristic colors. Colour change was dependent on the concentration and intrinsic AOC of the standard/sample, as well as the type of metal oxide. Fifteen polyphenols commonly found in food were successfully assayed, their response was employed to a direct AOC evaluation and to develop an antioxidants database. The performance of the sensor array was compared to the ORAC assay, no direct correlation was found. However, the colorimetric database developed, using a portable color reader, was able to identify and quantify the major polyphenolic constituents in green tea exracts.

### 2.4. Quantum Dots 

Quantum dots (QDs) are NMs that have also recently attracted large interest in analytical chemistry. QDs are semiconductor nanocrystals with size dependent fluorescent properties. Compared with the traditional organic fluorescence probes, QDs have many advantages, including broadband excitation, narrow bandwidth and high intensity emission [68]. QDs have become important as fluorophores in biological applications [69], because the possibility to size-tune fluorescent emission as a function of the core size, shape and material [45].

An antioxidant activity assay based on L-cysteine-capped CdTe QDs was proposed by Hemmateenejad et al. [58]. Measurement of the inhibitory effect of antioxidant/PCs (due to their scavenging activity) on the UV-induced bleaching of CdTe QDs was used. Quercetin, tannic acid, caffeic acid, gallic acid, naringin, trolox and four different types of tea reduced photobleaching effect induced by ROS. The sigmoidal curve obtained by plotting the relative inhibition of photobleaching vs. the logarithmic concentration of antioxidant/polyphenolic was employed to evaluate the AOC. The results were compared with the FC assay with a good correlation (R = 0.996). No comparison with AOC methods was reported. 

CdTe QDs were also used by Akshath et al. [59] for an indirect quantification of polyphenols, taking advantage of the enzymatic reaction of laccase. The latter convert polyphenols to mono/polyquinones that quench QDs fluorescence. This phenomenon of charge transfer from quinones to QDs was exploited as indirect polyphenol optical labels. A similar approach was adopted by Li et al. [70]. CdTe QDs emitting at 582 nm were synthesized and conjugated with the enzyme. Catechin, epicathechin and epigallocatechin-gallate, employed as model analytes, exhibited different quenching activity. The method was applied to detect polyphenols in spiked plant extracts with good recovery. No comparison with existing methods was reported. CdTe QDs fluorescence recovery in the presence of the analyte was proposed by Dwiecki et al. [60]. Polyphenols acted as electron donors for the CdTe–sodium periodate system, leading to perturbation of the transfer of excited electrons from QD to the acceptor molecules occurring in the absence of polyphenols. The fluorescence was measured at 564 nm with excitation at 530 nm. The fluorescence intensity increase was linearly dependent on the concentration of antioxidant used (catechin, quercetin, rutin, chlorogenic acid, gallic acid, caffeic acid). PCs in common drinks (coffee, tea, and herbal infusions) were successfully determined and compared well with FC. Because of the differences in reactivity of the standards, the method seems to estimate the AOC more than the phenolic content. 

Graphene (GR) QDs (GRQDs) were used by Benítez-Martínez et al. [61] for the polyphenol fraction evaluation of olive oil extracts. Blue GRQDs were obtained by pyrolysis of citric acid. The strategy was based on fluorescence quenching obtained by phenols with GRQDs via π-π stacking and non-covalent interactions. The GRQDs used in this work were flat circular nanosheets of average diameter 3.6 ± 0.9 nm. Oleuropein was able to quench the GRQDs fluorescence via a charge transfer mechanism in which GRQDs act as donor. The emission band (at 474 nm) undergoes a green shift when phenols from real samples are added, indicating a common charge transfer fluorescence quenching. In addition, higher quenching was observed when the polarity of the solvent tested increased, confirming a charge transfer quenching mechanism. The applicability of the method was evaluated on four different types of real olive oil samples and responses similar to the FC method were obtained. The non-specific interactions of polyphenols-GRQDs limits the use with other more complex foods matrices. Luminescent blue GRQDs were also used as sensing probes in a paper-based sensing device with a smartphone readout; proposed for AOC evaluation related to flavonoid content in wine samples [62]. GRQDs were added to waxed spots on nitrocellulose strips and 5 mm LED of 20 mA at 365 nm wavelength was used as UV source. 4–8 μL of the analytes/samples were added to the fluorescent sensing area in the paper strip, and, after drying at room temperature, pictures were taken with a smartphone. PCs caused different levels of GRQDs quenching (morin > myricetin > quercetin > kaempherol). The sensors resulted able to detect flavonoid structure. Wine samples of different vintages were investigated using this paper sensor, the results allow to relate the AOC to wine ageing. Despite the interesting results obtained, the ability to assess AOC by the device still needs to be proved. 

## 3. Electrochemical Strategies 

Electrochemical strategies are a versatile tool for fast and low cost analysis. Electrochemical approaches for the direct sensing of antioxidants as polyphenols or AOC of a sample provide the possibility to work directly in real samples for the majority of the cases. Indeed, different types of electrodes/transducers types and configurations can be used for the purpose in flow or stationary mode [71]. 

In 2011 Barroso [72] reviewed the electroanalytical approaches for the assessment of the total or individual AOC exploiting modified and unmodified electrodes, in drugs and some foods. Recently (2017), Hoyos-Arbeláez et al. [73] reviewed electrochemical methods as a tool for determining the AOC of foods and beverages. The review is focused on the classical electrochemical techniques, giving an historical overview on the electrochemical AOC assessment, without focusing on the particular contribution of NMs.

Direct electrochemical oxidation of polyphenols onto classical carbon-based materials is relatively easy to achieve being the oxidation potential needed dependent on the polyphenol structure. However, passivation, produced by the oxidation of phenols at the working electrode surface during the measurement, is a relevant drawback. The latter is due to the adsorption of oxidation products to form a non-compact monolayer on the surface of the working electrode [74]. NMs can help in reducing or eliminating the passivation of the electrode area in addition to the already reported ability of improving sensitivity (high surface/volume). At the same time unique catalytic properties produced by NMs shape and conformation, or by the use of nanocomposites can be exploited [75]. In general, NMs results in increased faradaic currents and shift the reaction towards lower (or higher) potentials for a wide variety of compounds, with respect to classical unmodified electrodes [76]. Furthermore, the NMs’ features can bring to an increase of the selectivity, allowing the assay of compounds with similar structure, but different electrochemical behavior.

Different nanocarbon allotropes may be used for sensing purposes, typical examples are fullerenes, carbon nanotubes (CNTs), carbon nanoparticles and graphene. They can be used in different structural configurations, with different oxidation degrees [77,78], functionalized with inorganic and organic molecules, coupled to polymeric structures or with other NMs (hybrid NMs). Ziyatdinova and Budnikov [21] have listed in 2015 different approaches to the use of NM-modified electrodes, mostly designed for the analysis of single PCs. In particular, the discussion was focused on the electroanalytical techniques used and their advantages and limitations. Different reviews have been reported on electrochemical methods for the evaluation of antioxidant compounds [26,79], or the use of NMs as sensing performance enhancer [75,76,80]. 

**Figure 2:**
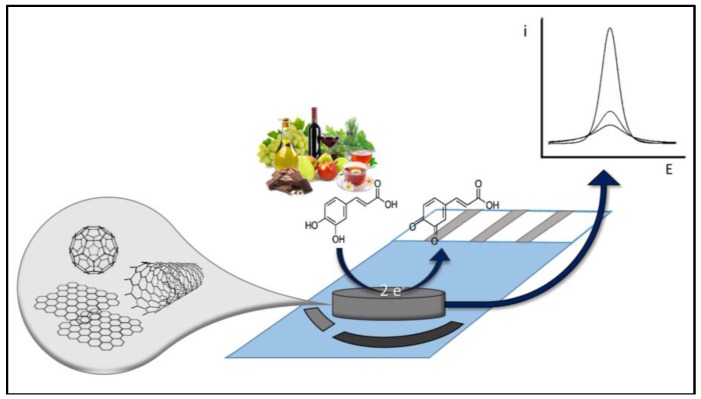
Schematic representation of polyphenols oxidation onto a nanomaterial based electrode.

However, none of these works focused particularly on the use of NM-based sensors for the direct electrochemical detection of food polyphenols and for evaluation of their AOC; this will be the subject of the following section. Figure 2 schematize a generic polyphenols reaction onto NM-based electrodes. Table 2 summarizes the main features of the reviewed NM-based electrochemical strategies.

### 3.1. CNTs

Since their discovery CNTs have drawn considerable research attention and have shown great potential application in many fields due to their unique structural, mechanical, and electronic properties [81], their role on sensing strategies were reviewed by different authors [81,82,83].

For the detection of polyphenols Lismery et al. [84] proposed a simple modification of glassy carbon electrode (GCE) surfaces with single-walled carbon nanotubes (SWCNTs). CV of gallic acid resulted in two anodic peaks, with a relevant decrease in the overvoltage and increase in the anodic peak values for the GCE-SWCNTs, suggesting an improved electron-transfer kinetics. The gallic acid oxidation peak observed at +0.35 V (vs. Ag/AgCl) using the DPV was used to build a calibration curve and to quantify the antioxidants. A detection limit of 3.0 × 10^−7^ mol L^−1^ of gallic acid was achieved. The total amount of red and white wine polyphenols was estimated using the gallic acid calibration curve and a good correlation was found with FC (R = 0.980). The reactivity of polyphenols commonly found in wine is not reported, however, it is relevant that glucose and ascorbic acid at the typical concentrations found in wines did not interfere with the measurement.

Arribas et al. [85] proposed a strategy for TP assessment in wine using GCE modified with multi-walled carbon nanotubes (MWCNTs) and measurement in flow injection analysis (FIA). GCEs were modified via drop casting, using different CNTs dispersions added with Nafion or polyethylenimine (PEI). The electrochemical evaluation of different polyphenols (gallic acid, caffeic acid, ferulic acid, *p*-coumaric acid) resulted in a decreased overpotential for their oxidation for all the MWCNTs modified electrodes.

However, the MWCNTs/PEI electrode gave the higher oxidation currents for all the compounds and was assembled in a FIA system for the measurement in real samples. Two electrochemical indexes were proposed: measuring samples at +0.3 V for easily oxidizable polyphenols (correlated to compounds with high antioxidant capacity, mainly *o*-diphenols) and at +0.70 V for TP. Response was linear for the four model analytes in the concentration range from 1.0 × 10^−7^ to 1.0 × 10^−4^ mol L^−1^ and limit of detections (LODs) using the two detection potentials, were below 0.1 × 10^−6^ mol L^−1^. No fouling of the electrodes was observed after the assay of 15 samples of wine at both potentials; as expected, better correlation with FC assay was found for the assay at +0.7 V (vs. Ag/AgCl) with respect to +0.3 V (vs. Ag/AgCl). Selective electrochemical detection of *o*-phenolic compounds was obtained also for olive oil extracted phenols [64]. Graphite screen printed electrode (SPE) modified with an inorganic mediator, Na_2_MoO_4_ and SWCNTs and MWCNTs bearing amino or carboxylic groups were used. CNTs in this case were used to maximize the retention of the molibdate mediator onto the electrode. The SPE-Mo/MWCNTs-NH_2_ electrode gave the best response for hydroxytyrosol (*o*-diphenol) and tyrosol (*m*-phenols) allowing a selective detection. An amperometric FIA system was used to assess the amount of *o*-diphenols of 13 olive oils samples. Using cathecol as referring standard the analytical range obtained for *o*-diphenols was 0.2–50 mg L^−1^; this was wider than the spectrophotometric method used for comparison (10–60 mg L^−1^).

A chronoamperometric estimation of cognac and brandy AOC using MWCNTs drop casted on GCE has been reported by Ziyatdinova et al. [86]. Voltammetric (DPV) behavior of the main antioxidant constituents of cognac (ellagic and gallic acids, syringaldehyde, coniferaldehyde, vanillin, 5-hydroxymethylfurfural and furfural) has been investigated. Ellagic acid, being the main antioxidant of cognac, was used as reference. AOC of different brands of cognac and brandy was investigated and compared with the FC, DPPH•, constant-current coulometry with electrogenerated titrants and FRAP assays. The correlation coefficients obtained (R = 0.913–0.970) confirmed that the method reflects antioxidant contents in cognacs and brandies. The same electrode was successfully employed for the AOC assay of coffee [87] and wine samples [88]. In both cases, the electrochemical behavior of the main polyphenols present in the food matrix were studied. On the basis of the data obtained AOC indexes were proposed. For coffee beans and instant coffee samples DPV on GCE-MWCNTs was employed and the results have been expressed as chlorogenic acid equivalents. Positive correlation was observed with coulometric titration with electrogenerated hexacyano-ferrate(III) ions (R = 0.960) analyzing a total of 16 samples. A chronocoulometric method was developed for the evaluation of the AOC of wine. A one-step chronocoulometry at +0.83 and +1.18 V was performed with the GCE-MWCNTs electrodes for red and white wines, respectively. Positive correlations were observed with coulometric titration with electrogenerated bromine (T = 0.8957 at *n* = 5 and R = 0.8986 at *n* = 4 for red and white wines, respectively). No data on reproducibility and/or fouling of the electrodes were reported.

Very recently, Eguílaz et al. [89] modified a GCE with SWCNTs covalently functionalized with polytyrosine (GCE-SWCNT/Polytyr) for the determination of total polyphenolic content in tea extracts. The electrode was obtained casting ethanol/water SWCNT-Polytyr dispersions. GCE-SWCNT-Polytyr resulted in a decreased oxidation overvoltage and an enhancement of the oxidation peaks current for all the polyphenols tested. The GCE-SWCNT/Polytyr was used to quantify the TP in different tea samples; no comparison with other methods is reported. 

Crevillén et al. [90] integrated SPE-MWCNTs into a microfluidic platform (glass ‘double T’ microfluidic chip with a 74 mm long separation channel) for food polyphenols analysis. Microchip technology has become an attractive alternative to the well-established conventional separation techniques; this is primarily due to the ability to analyze small volumes of sample, speed of analysis, reduced cost and waste, and portability. Electrokinetic microchips integrated with SPE-MWCNTs as detectors were proposed for the determination of total isoflavones and detection of antioxidant profiles. To this aim two analytical formats, a flow injection and a separative electrokinetic driven system using real samples were exploited. Reference materials and dietary supplement were successfully analyzed with the FIA system and the concentration of total isoflavones in the samples was calculated as genistein equivalents. The values obtained were very close to those declared by the manufacturer. The separation mode in the microchip was employed with apple and pear extracts. Under optimized conditions (borate pH 9, 50 mM), five natural antioxidants (arbutin, phloridzin, (+)-catechin, rutin and ascorbic acid) were separated in less than 250 s. Different sensitivities and LODs for sample polyphenols quantification were obtained for the different antioxidants assayed, in all the cases they were satisfactory. The use of MWCNTs allowed the improvement of selectivity increasing the number of theoretical plates and resolution, because the faster electron transfer increase the peaks definition and reduce their broadening. Recoveries were acceptable with values ranging between 70 and 120% for all antioxidants. Press-transferred SWCNTs electrodes were coupled to microfluidic chips to develop a class-selective electrochemical isoflavone index by Vilela et al. [91]. A qualitative and quantitative assessment of isoflavones based on the co-migration of the total glycosides (TG) and total aglycones (TA) was proposed, and applied to soy extracts. The electrophoretic separation was performed at a ‘double T’ glass chip, and the pH of running buffer was established at 9.0 (pH > pKa for all isoflavones involved). The separation of isoflavone antioxidant classes of glycosides and aglycones was achieved in less than 250 s. Qualitative assessment of isoflavone indexes towards detection of TA and TG in soy samples was exploited. The data were in agreement for migration times obtained for TG and TA present in the samples with respect to standards.

### 3.2. Carbon Black

Carbon black (CB) is a carbon material widely used as reinforcing material and as a filler in the preparation of rubber and plastic compounds and composites. It is characterised by a primary structure constituted of spherical particles with diameters between 30 and 100 nm and a secondary structure formed by aggregates having dimensions in the range of 100–600 nm [103]. CB dispersions after sonication, in appropriate solvents, appears as carbon nanoparticles. The ‘nano CB’ exhibit excellent conductivity, unique electrochemical properties and cost-effectiveness (about 1 euro/kg). For this reasons, in the last years several works have been reported on the CB dispersion for electrode modification, indicating remarkable electrocatalytic properties towards several species of analytical interest. Silva et al. [104] discussed the use of CB in electrochemical biosensors and Arduini et al. [105] the use of the same devices for pesticides detection. 

Talarico et al. [92] modified SPE with CB for the detection of different PCs, commonly found in food. The sensor was constructed by drop casting CB on the surface of a SPE working electrode. The electrochemical behavior of SPE-CB towards catechol, tyrosol, caffeic acid, and gallic acid was studied. The data demonstrated an improved resistance to electrode fouling, in oxidation current and in peak-to-peak separation. Calibration curves obtained via SQW gave limits of quantification (LOQs) of 10^−7^ mol L^−1^, 10^−6^ mol L^−1^, 0.8 × 10^−6^ mol L^−1^, and 2 × 10^−6^ mol L^−1^ for catechol, gallic acid, caffeic acid, and tyrosol, respectively. For mixtures of the compounds the SPE-CB electrode gave two clear oxidation peaks for *o*-diphenols and *m*-phenols at +0.1 V and +0.6 V (vs. Ag/AgCl). These data proved that CB is a nanomaterial exploitable for the detection of polyphenols. Detection of *o*-diphenols and monophenols in real samples was achieved by Della Pelle et al. [93] using a lab-made carbon black nanoparticles (CBNPs) press-imprinted transducer. The fabrication and characterization of the press-produced device, previously reported in detail [106,107], was obtained filtering and transferring by pressure, onto polymethylmetacrylate (PMMA), 0.5% (*w*/*v*) CBNPs in 1,2-dichloroethane. Two well-defined redox couples were found for *o*-diphenols such as hydroxytyrosol with oxidation potential at +0.27 V and reduction potential at −0.02 V vs. Ag/AgCl. The oxidation peak of the *m*-phenol tyrosol was found at potentials of +0.56 V (vs. Ag/AgCl); very low or no reduction peak was observed. Surface fouling was negligible considering the detection in DPV. The anodic peaks of *o-*diphenols and *m*-phenols using extracts from olive oil were in the range 0.12–0.16 V and 0.59–0.61 V (vs. Ag/AgCl), respectively. 

Raymundo-Pereira et al. [94] have exploited the synergy between Printex L6 nano-carbon black and AgNPs for the estimation of TP in wine samples. The silver nanoparticles were synthesized directly on CB using ethylene glycol as a reducing agent. A film coating layer was prepared on a GCE electrode surface by casting 6.0 µL of a CB-AgNP suspension in dimetylformamide (DMF). In order to prove the analytical performance of the electrodes toward PCs, DPV was applied to study the electrochemical behaviour of gallic acid. Oxidation current recorded using the CB-AgNP was higher than CB. A calibration curve was obtained in the 5.0 × 10^−7^ and 8.5 × 10^−6^ mol L^−1^ concentration range and the LOQ was 66.3 × 10^−9^ mol L^−1^. Glucose, ethanol and sulfite ions, studied as possible interferents, did not significantly influence the detection. Different real wine samples were analyzed with the proposed device and compared with a 4-aminoantipyrine-based spectrophotometric method. The phenolic content determined by electroanalytical methods was lower than that obtained by the spectrophotometric method, but the correlation was very good (R = 0.998). 

### 3.3. Graphene

Undoubtedly one of the most influent discoveries of the last decade was graphene (GR). GR is a two dimensional (2-D) sheet of carbon atoms linked via sp^2^ bonds. This configuration provides this material with extraordinary properties such as high conductivity and electron mobility at room temperature, low energy dynamics of electrons with atomic thickness, robust mechanical behavior and flexibility. This material opened new gates in different scientific communities, and particularly in the analytical/electrochemical field. An overview of the use of this material on the electrochemical sensors was given by Gan in 2011 [108]. The author underlined the potential of this emerging two–dimensional nanomaterial, and its tremendous potential for electrochemical catalysis and biosensing. Martín et al. [109] have reviewed the GR terminology and properties, synthesis and characterization processes to obtain not only ‘‘true’’ graphene but also some chemical variants, such as graphene oxide, reduced graphene oxide and graphene nanoribbons.

The GR state has a deep influence on its electrochemical behavior, for these reasons different type of dopants and type of GR have been studied for the quantification of antioxidant activity [95]. Hui et al. [95] studied the electrochemical oxidation of gallic acid using undoped (GR), boron-doped (B-GR) and nitrogen-doped (N-GR) thermally reduced graphene deposited on a GCE. The materials were doped at high (H) or lower (L) concentrations. The oxidation current increased in the order: N-GR-L < GR < N-GR-H < B-GR-L < B-GR-H. The result was inconsistent with the level of structural defects of the nanomaterial. In fact, undoped GR, showed the largest density of defects. The greater electroactive surface together with the interactions between the electron withdrawing boron and the electron-donating oxygen atoms in gallic acid were supposed to play a crucial role by the authors. Using DPV, tea samples were assayed with the B-GR-H. Peak height of gallic acid was unaffected by the increase in concentration of a potential interfering compound like ascorbic acid. 

Different outputs were obtained studying the electrochemical response of cathechins [79]. Comparison of GCE, boron-doped diamond (B-DD), thermally reduced graphene (T-GR), boron- doped graphene (B-GR) and nitrogen-doped graphene (N-GR) electrodes resulted in the best analytical performances for T-GR. The authors explained the data considering the lowest content of oxygen functionalities, the highest amount of structural disorders and the largest electroactive surface area. Thus, in this case, material properties play a major role towards the oxidation of catechin rather than the nature of heteroatom used for the doping. However, both works [95,96] are much more focused on the demonstration of the oxidation mechanism than the application on real samples.

An electrochemical sensor based on a Fe_2_O_3_/electro-reduced graphene oxide composite was employed for the estimation of the AOC in wine [97]. The electrode (GCE-GR reduced-Fe_2_O_3_/Chit ) was modified via drop casting on GCE using the hybrid material of chitosan (CS), fishbone-shaped Fe_2_O_3_ (fFe_2_O_3_), and electrochemically reduced GRO. Reduced graphene was demonstrated to have improved electrocatalytic proprieties vs. oxidized graphene in the nanocomposite for the oxidation of gallic acid. Using DPV a good linear relationship of peak height vs. log of concentration was achieved in the 10^−6^–10^−4^ mol L^−1^ range; a LOD of 1.5 × 10^−7^ mol L^−1^ was reported. A standard addition procedure for the determination of polyhenols in a wine sample gave data reasonably related to HPLC, being the content estimated by the sensor higher. Glycerol, sorbitol, amino acids (glycine, glutamic acid and lysine), citric acid, glucose, caffeic acid, and ascorbic acid had no significant influence on the detection of gallic acid. A nanocomposite made by graphene/poly (3,4-ethylenedioxythiophene):poly(styrene sulfonate) electrosprayed on a modified screen-printed carbon electrode (SPE-GR/PEDOT/PSS ) has been reported by Tirawattanakoson et al. [98]. After optimization of the nanocomposite and morphological characterization, the electrochemical characterization revealed larger area vs. SPE, as expected, and also faster electron transfer kinetics of the probe. The nanocomposite sensor has been used for the indirect evaluation of AOC, replacing the classical spectrophotometric measurement of DPPH• with the developed electrochemical approach. A calibration curve for Trolox was obtained in the 5.0 × 10^−6^–3 × 10^−5^ mol L^−1^ range with a LOD of 0.59 × 10^−6^ mol L^−1^. The AOC of five Thai herb and herbal beverage samples was calculated and compared with the spectrophotometric measurement. The results obtained were not significantly different between the two methods. No mention of the direct oxidation of PCs is given in the paper. 

### 3.4. Alternative Strategies Based on Nanomaterials 

A carbon paste electrode (CPE) modified with a core-shell hybrid NM constituted by surface active maghemite nanoparticles (core) and tannic acid (shell) was used for the determination of polyphenols in blueberry extracts [99]. Tannic acid is a well-known chelator of iron(III) due to the combined action and the vicinity of hydroxyl groups on the aromatic rings. The presence of tannic acid led to an increase of peak currents, a decrease of the peak separation, indicating an improved reversibility of the redox reactions, and, at the same time, reduction of the resistance. The carbon paste electrode has been used for the electrochemical detection of hydroquinone and blueberry extracted samples by SWV. Comparable data with the FC were obtained.

Andrei et al. [100] proposed a disposable SPE based on ‘biomimetic’ (oxidase-like activity) cerium (IV) oxide NPs (nanoceria) for the detection of wine phenolic antioxidants. The use of nanoceria in the sensor design enables oxidation of PCs, particularly those with *o*-diphenol functionalities, to their corresponding quinones that are detected at the SPE surface. The sensor was realized casting a nanoceria dispersion onto a SPE, and the electrochemical mechanism studied by gallic acid CVs. Amperometric detection at −0.1 V (vs. Ag/AgCl) was employed to assess the reactivity of gallic acid, caffeic acid, quercetin, ascorbic acid and epicatechin. The gallic acid linear range was between 2 × 10^−6^ and 2 × 10^−5^ mol L^−1^ with a LOD of 1.5 × 10^−6^ mol L^−1^. In the applied conditions the electrode was selective vs. glucose, tartaric acid, ethanol, citric acid, sulphur dioxide. Data obtained on wine samples were compared with the ABTS• assay; no correlation was found among the results provided by the two methods. The authors concludes that the device can be potentially included in an e-tongue device for an analytical fingerprinting, because an information different from ABTS• is given.

Graphitized mesoporous/chitosan modified glassy carbon electrodes were employed for tea polyphenols assessment using flow injection analysis coupled with a dual electrochemical detector [101]. Catechin, epicatechin, epicatechin gallate, epigallocatechin and epigallocatechin gallate, which are made by combination of 1,2-dihydroxybenzene, 1,3-dihydroxy- and 1,2,4-trihydroxybenzene were studied because they are commonly found in tea. Epigallocatechin gallate is the compound with the highest antioxidant capacity. The graphitic nature of mesoporous carbon materials and its chemical interaction with chitosan was proved using Raman spectroscopy and transmission electron microscopy (TEM). The FIA electrochemical detector was set at two different potentials, +0.1 and +0.7 V (vs. Ag/AgCl) and the phenolic standards analyzed in the 10^−7^–10^−5^ mol L^−1^ range. Analytical validation of the approach was tested by analyzing the polyphenolic functional groups in nine different commercial tea samples; recovery values ~100% were obtained.

Cetó et al. [102] reported the application of a (bio-)electronic tongue system composed by an array of epoxy–graphite modified electrodes, to assess phenolic antioxidants in wine. The aim of the work was the simultaneous determination of the different polyphenols, using different redox enzymes (tyrosinase and laccase) and copper nanoparticles entrapped in the epoxy resin. An artificial neural network was employed for building the response model, considering 23 different current intensities taken at specific potentials extrapolated by the CVs standard PCs. 10 wine samples were spiked with variable amounts of three standard PCs randomly distributed; good recovery yields (values of 104%, 117% and 122% for catechol, caffeic acid and catechin, respectively) were obtained. Complexity of the PCs present in samples and of the prediction model makes this approach not straightforward for on-site analysis.

## 4. Nanomaterial-Based Enzyme Electrodes

In the development of biosensors, the biological element, integrated into a transducer, allows the selective recognition of the target analyte or class of molecules. For the detection of polyphenols, for example, Del Carlo et al. [110] recently proposed the use of a short cyclic peptide designed in silico for the selective recognition of chlorogenic acid. Also DNA based sensors have been used for AOC assay. This is an indirect assay since it measures the protective action of PCs vs. damage induced to DNA by ROS. However, few papers are food oriented or use NMs. In this respect it is worth to mention a MWCNTs DNA-biosensors for the AOC evaluation of rutin and tea extract proposed by Ziyatdinova et al. [111]. The sensor was functionalyzed by double stranded calf thymus or herring sperm DNA deposited on the surface of a MWCNTs SPE. The SPE-MWCNTs/DNA was constructed with a layer-by-layer coverage and a impedimetric strategies were employed to assess the DNA damage, using [Fe(CN_6_)]^3−^ as redox probe.

**Figure 3:**
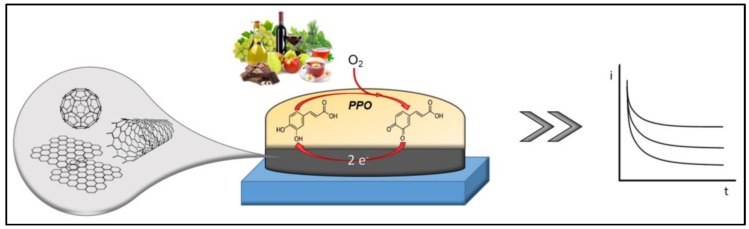
Schematic representation of polyphenols (caffeic acid) detection onto a nanomaterial based enzyme (polyphenols oxidase) sensor.

However, enzymes electrodes attracted the largest interest in this area because of the analytical potential of coupling selectivity and catalytic action. Nanomaterials offers unique and creative configurations for enzyme electrodes realization integrating the already mentioned promotion of the electrochemical reaction with the possibility of direct wiring of enzymes to the electrode surface [112]. Classical enzyme electrodes for polyphenols are based on polyphenol oxidase (PPO). PPO has copper as prosthetic group and uses molecular oxygen as a co-substrate. It catalyzes two reactions: the first is a cresolase activity, adding a hydroxyl group to a monophenol at the *ortho* position to convert it into an *o*-diphenolic compound. The second, known as catecholase activity, converts the diphenolic compound into quinone [113]. Based on substrate specificity and mechanism of action, PPOs can be classified in different types: tyrosinase, catechol oxidase and laccase. Indeed, PPO catalyzes selectively the oxidation of monophenols by molecular oxygen to form *o*-diphenols, which are then oxidized to *o*-quinones. Generated quinones can be reduced at low potential onto the transducer surface producing a current signal proportional to the phenolic compound. Furthermore, the *o*-diphenols generated can be re-oxidized by the enzymes creating an enzymatic/electrochemical amplification cycle. Figure 3 schematize a generic polyphenols reaction onto a NM-based enzyme sensor. Table 3 summarizes the principal features of the reviewed NM-based enzymatic electrochemical strategies.

Recently Rodríguez-Delgado et al. [123] and additionally Gul et al. [113] reviewed laccase- and PPO-based biosensors, for the detection of PCs in industrial waste waters and for industrial applications, respectively.

The ability of laccases to oxidize a broad range of PCs is employed in the majority of the biosensors for the detection of polyphenols. In this section, recent application of nanomaterial based enzyme electrodes for the detection of polyphenols in food is reported. The majority of the papers exploit the features of CNTs because they have been the first carbon-based nanomaterial used for electrochemical sensors and also because they can be easily functionalized, creating bonds with the enzymes or other sensors components.

Diaconu et al. [114] developed a laccase–MWCNT–chitosan biosensor for the TP content evaluation of two in vitro cultivated plants: *Salvia officinalis* and *Mentha piperita*. Laccase from *Trametes versicolor* (an enzyme with broad substrate specificity for the phenolic substrates) was immobilized in a single step by entrapment into the nanocomposite film during the electrodeposition of MWCNT and chitosan. The electrodeposition of MWCNT/CS film on gold electrode surface leads to a two times increase in current intensity. Calibration of the laccase biosensor was performed with four phenolic acids (caffeic acid, chlorogenic acid, gallic acids and rosmarinic acid) at −0.2 V (vs. Ag/AgCl); the response time of the electrode was 2 min. The widest linear range was obtained for caffeic acid and rosmarinic acid, with a limit of detection of 1.51 × 10^−7^ mol L^−1^ and 2.33 × 10^−7^ mol L^−1^, respectively. The sensitivity of the biosensor in the micromolar concentration range was supposed to be caused by MWCNTs incorporation into the chitosan layer. The enzyme electrode was applied to evaluate the TP of in vitro cultivated *S. officinalis* and *M. piperita* extracts; data were reproducible and have been expressed in rosmarinic acid equivalents. The stability was checked with RSDs of 5.6% and 10.0% for 10 and 15 consecutive measurements, respectively. No comparison with other methods is reported. 

A similar approach was carried out by Fusco et al. [115] using SPE -SWCNTs-modified electrodes and polyazetidine to immobilise laccases from *Trametes versicolor* and *Trametes hirsuta*. The developed enzyme electrodes were employed in fixed potential amperometry (−0.1 V vs. Ag/AgCl) FIA; similar affinity and sensitivity toward gallic acid was obtained for the two laccases. Polyphenol indexes for twelve wines (six white and six red) was calculated and compared with FC. A slight underestimation of the polyphenols content was achieved with the developed method. Sulphur dioxide, reducing sugars and ascorbic acid did not interfere with the biosensor. The developed biosensors resulted to be usable for six measurements without any evident loss of performance.

A nanocomposite constituted by gold electro-C-MWCNT-polyaniline/AgNPs was studied for the immobilization of laccase by Rawal et al. [116]. MWCNTs have been initially carboxylated in acidic solution and mixed with aniline and, later, the mix electrodeposited onto Au electrode. AgNPs prepared with sodium borohydride were deposited onto the modified Au electrode before covalent immobilization of laccase from *Ganoderma* sp. Under optimized conditions, the chronoamperometric (+0.22 V vs. Ag/AgCl, reading the signal at 6 s) detection of guaiacol was linear in the 1.0 × 10^−5^–5.0 × 10^−4^ mol L^−1^ range with a detection limit of 5.0 × 10^−8^ mol L^−1^. The enzyme electrode was employed for the determination of polyphenols in five commercial brands of tea leaves, alcoholic beverages, and pharmaceutical samples. A good correlation with FC assay (R = 0.990) was obtained. Negligible interference was observed for ascorbic acid, glucose, fructose, and citric acid. Remarkably, the biosensor was very stable (20% decrease of the signal after 200 measurement carried out in four months). The same enzyme electrode was used by Chawla et al. [117] substituting AgNPs with NiNPs. Similar analytical performances were obtained in terms of sensitivity, stability and measurement of polyphenols in real samples (fruit juices).

Graphene oxide (GRO) mixed with MWCNTs was used to realize tyrosinase and laccase enzyme electrodes for the TP estimation of juice samples [121]. GRO and MWCNTs were casted on a GCE, and followed by GRO electrochemical reduction. The enzymes (Laccase from *Trametes versicolor*, tyrosinase from mushroom) were deposited and three materials were investigated, chitosan, Nafion, and bis(trimethylsilyl)acetamide (BSA) crosslinked with glutaraldehyde, as immobilizers. The electrochemical performance of the GCE was highly improved when GRO and MWCNTs were in the 1:1 (*w*/*w*) ratio. The best immobilisation procedure was BSA-glutaraldehyde for laccase, and the chitosan based for tyrosinase. Chronoamperometric curves recorded at 0 V and −0.1 V vs. Ag/AgCl for pyrogallol, epicatechin, gallic acid, 1,2-dihydroxybenzoic acid, caffeic acid, chlorogenic acid, rutin, catechin and dopamine exhibited higher sensitivity for the laccase enzyme electrode. Analysis of fruit juices, expressed as epicatechin equivalents gave very similar results for the two enzyme electrodes and compared favorably with the ABTS• assay. Reduced graphene oxide and suspensions of PtNPs were used to modify a SPE and realise the disposable laccase enzyme electrode proposed by Eremia et al. [118]. Laccase from *Trametes versicolor* was drop casted and covered with a Nafion film onto the modified SPE. Amperometric (at +0.1 V vs. Ag/AgCl) detection of caffeic acid gave a very good limit of detection of 9.0 × 10^−8^ mol L^−1^. A Michaelis–Menten apparent constant of 2.75 × 10^−6^ mol L^−1^ was obtained, indicating, surprisingly, improved affinity of immobilised laccase towards caffeic acid. The sensitivity was in this order: caffeic acid > gallic acid > luteolin > rosmarinic acid. Analysis of tea infusions gave good correlation with FC method (R = 0.996). 

A multilayer enzyme electrode consisting in AuNPs, fullerene and *Trametes versicolor* laccase onto a gold electrode surface was proposed by Lanzellotto et al. [119], and applied to assess the TP content of wine samples. Gallic acid response, measured in FIA at −0.1 V vs. Ag/AgCl was linear in the 3.0 × 10^−5^–3.0 × 10^−4^ mol L^−1^ range with a LOD of 6.0 × 10^−6^ mol L^−1^. The enzyme electrode gave a slight overestimation of polyphenolic content with respect to FC. After 120 analysis the enzyme electrode still retained 87% of the initial activity. Molybdenum disulfide (MoS_2_) nanoflakes and graphene quantum dots (GRQDs) were used to modify SPE and realize a laccase enzyme electrode by Vasilescu et al. [120]. It was hypothesized that laccase immobilization at the surface of the electrode occurs through electrostatic interaction between the negatively charged laccase and the positively charged GRQDs. Chronoamperometric measurements at +0.05 V (vs. Ag/agCl) gave good sensitivity for chlorogenic acid, caffeic acid and epicathechin. Analysis of eight wines gave data comparable to the FC method and no loss of reactivity was observed for seven continuous measurement. 

Nadifiyine et al. [122] proposed a tyrosinase carbon black paste electrode for TP determination in olive oil. The tyrosinase (extracted from mushrooms) was immobilized by cross-linking using a BSA glutaraldehyde procedure. High sensitivity for cathecol detection was achieved with a LOD of 6.0 × 10^−9^ mol L^−1^. Twenty different PCs were tested in the whole range of linearity. The enzyme electrode exhibited high sensitivity toward hydroxytyrosol and tyrosol; the major PCs in olive oil. The results obtained with this enzyme electrode overestimates the concentration of phenolic content obtained using FC for olive oil extracted samples; however, a good correlation coefficient was obtained R = 0.939. The electrode response maintained its initial value for three weeks. 

## 5. Conclusions and Perspectives 

The aim of this review was to provide a critical state of the art on the most recent developments of new NM-based tools and strategies for TP determination and AOC evaluation (derived from polyphenols) in foodstuffs. The study of antioxidant compounds continues to be a very challenging field, because of the implications of these compounds in particular pathologies and their importance in food stability; the polyphenol area of research seems to be an infinite source of discovery and exciting new achievements. In the past years, and also today, the development of methods to study these relevant phytochemicals, continues to be of great interest in the food analysis, chemistry and technology community. In this respect, the lack of official methods can generate confusion; however, this is unavoidable because of the multiple of mechanisms of action of antioxidant compounds (or their natural endogenous mixture). Thus, a single method cannot cover all the desired functional proprieties that need to be assessed for a particular purpose.

The most used screening method for the detection of TP is FC while different methods are generally used for the assessment of AOC (ABTS•, FRAP, etc.). The use of sensors and sensing strategies, alternative to accepted methods, can be implemented in case they have a clear analytical advantage or they provide new information. As an example, classical electrochemical methods can be used for reducing the use of reagents and with very rapid time of analysis. On the other hand, problems linked to electrode fouling should be taken in consideration because of the heterogeneous nature of the reaction. At the same time electrochemical methods can provide a certain degree of selectivity making possible the selection of different classes of compounds (i.e., *o*-diphenols vs. monophenols) giving important information about foods stability (shelf-life). The ‘tunable’ and unique features of nanomaterials, the possibility to combine different types of nanomaterials, the easy way to functionalize them, give more options and possible advantages over existing methods; particularly, increased sensitivity, selectivity and long term stability of the sensing systems. These contribute to make the analytical screening assays more robust and informative for the users. The possibility of coupling enzymes to the nanostructured electrodes, confers more complexity to the system, but can improve the sensitivity and drive the selectivity towards the biological element rather than the electrochemical method. In this case the key parameter is stability (life time) of the enzyme electrode. Looking at the reviewed papers, a lot of the works have been directed to the “proof of concept” of the use of the sensor and, for this reason, the food matrices analyzed have been easily extractable fractions in water, or infusions, with high content of phenols. In this sense very few papers have been directed to run a proper validation with samples of different nature and different phenolic content. 

The use of MNPs with optical detection, is intrinsically less sensitive that the electrochemical approach. However, it allows the evaluation of TP and/or AOC in a rapid way and can be alternative to the most used methods. In fact, for some of the reported methods, the simplicity of the assay conjugated with the possibility of quantification based only on a colorimetric scale is noteworthy. The advantage relies on the possibility, for some of the developed assay, to work without extraction from the samples (e.g., for olive oil), or with a simple extraction procedure, as well as directly on paper devices. This approach appears promising but more studies directed to improve the selectivity using different MNPs and procedures are needed. This will open, in our opinion, definitively new perspectives for the use of this approach directly on site.

In conclusion it is clear that a remarkable effort of the recent research has been directed to the development of new sensing strategies useful for TP or AOC assessment and that nanomaterials can play a crucial role in implementing this new approaches into the real world. More research work is expected in the next years both for validation of the procedures on different real samples and for the development of new nanomaterial based sensing tools.

## Figures and Tables

**Table 1:** Main strategies and features of optical nanomaterial-based assays for food polyphenols evaluation.

Work Aim	Nanomaterial	Food Matrix	Sample Pretreatment	Method Principles	Detection Mechanism	Ref.
AOC samples comparison	AuNPs	Fruit extracts	LLE	Au reduction, mild condition	Optical: LSPR	[47]
AOC new index proposal/sample evaluation	AuNPs	Olive oil	SPE	Au reduction, mild condition	Optical: LSPR	[46]
TP new index proposal/sample evaluation	AuNPs	Olive oil, Chocolate	No required (sample dilution in DMSO)	Au reduction in fat matrix, DMSO strategic solvent	Optical: LSPR	[48]
AOC samples evaluation	AuNPs	Rapeseed	LLE	Au reduction at pH 4.6	Optical: LSPR	[49]
AOC paper based sensor realization	AuNPs	Tea, Wine	Infusion/No required	Au reduction on paper	Visual, Optical (LSPR)	[44]
AOC sample evaluation	AgNPs	Fruit juices, Olive Oil	Infusion/LLE	AgNPs seed-growth	Optical (LSPR)	[50]
AOC sample evaluation	AgNPs	Ginger	LLE	AgNPs seed-growth	Optical (LSPR)	[51]
AOC samples evaluation	AgNPs	Rapeseed	LLE	Ag reduction, 1 h	Optical (LSPR)	[52]
AOC evaluation	Ceria NPs	Teas, medicinal mushrooms	Infusion	Ce(IV) reduction and ceria NPs colour inhibition, both on paper	Visual, Optical (LSPR)	[53]
AOC sample evaluation	Cerium oxide NPs	Rapeseed and its By-Products	LLE	Ce(IV) reduction, mild condition 60 min	Optical (LSPR)	[54]
AOC sample evaluation	Iron oxide NPs	Rapessed oil	LLE	Fe (III) reduction, mild condition 50 min	Optical (LSPR)	[55]
AOC and total catechins evaluation	Rhodium NPs	Teas	Infusion	RhNPS LSPR shifting	Optical (LSPR)	[56]
Polyphenols evaluation	Ti(IV)oxo, ZnO, SiO_2_, ZrO_2_, TiO_2_, Fe_2_O_3_, CeO_2_ nanoparticles	Green Tea	Infusion	MNPs-oxides surface complexation with polyphenols	Visual color changes	[57]
TP estimation	CdTe QDs	Teas	Infusion	CdTe QDs fluorescence quenching inhibition	Fluorescence	[58]
Fluorescence	CdTe QDs	Plant extracts	LLE	CdTe QDs laccase mediated/fluorescence quenching	Fluorescence	[59]
TP estimation	CdTe–sodium periodate	Tea, Herbal infusions	Infusion	CdTe QDs fluorescence quenching inhibition	Fluorescence	[60]
TP estimation	Graphene QDs	Olive oil extracts	LLE	Graphene QDs quenching	Fluorescence	[61]
AOC estimation	Graphene QD	Wine samples	Dilution	Graphene QDs quenching on paper	Fluorescence	[62]

**Table 2:** Electrochemical sensors based on nanomaterial for food polyphenols evaluation: main features and strategies.

Work Aim	Nanomaterial	Food Matrix	Sample Pretreatment	Method Principles	Detection Mechanism	Linear Range/LOD	Ref.
AOC sample evaluation	SWCNTs	Red/white wine	No required (sample dilution)	Polyphenols ox. at GCE-SWCNTs	DPV	Gallic acid: 5.0 × 10^−7^ to 1.5 × 10^−5^; 3.0 × 10^−7^ mol L^−1^	[84]
TP estimation	MWCNTs	Red/white wine	No required (sample dilution)	Polyphenols ox. at GCE-MWCNTs	FIA-amperometric	Phenolic acids: 1.0 × 10^−7^ to 1.0 × 10^−4^ mol L^−1^	[85]
*o*-Diphenol evaluation	MWCNTs-NH^2^-Mo	Olive oil	SPE	*o*-diphenols selective oxidation at SPE-Mo/MWCNTs-NH_2_	FIA-amperometric	Catechol: 0.2–5.0 × 10^1^ mg L^−1^	[64]
AOC evaluation	MWCNTs	Cognac and brandie	No required (sample dilution)	Polyphenols ox. at GCE-MWCNTs	Amperometry	Ellagic acid: 0.7 × 10^−6^–5.3 × 10^−5^ mol L^−1^; 0.2 × 10^−6^ mol L^−1^	[86]
AOC evaluation	MWCNTs	Coffee beans and infusions	Infusion	Polyphenols ox. at GCE-MWCNTs	DPV	Chlorogenic acid: 1.0 × 10^−6^ – 1.1 × 10^−3^ mol L^−1^; 0.21 × 10^−7^ mol L^−1^	[87]
AOC evaluation	MWCNTs	Red/white wine	No required (sample dilution)	Polyphenols ox. at GCE-MWCNTs	Chronocoulometry	Gallic acid: 5.2 × 10^−7^–2.9 × 10^−4^ mol L^−1^; 1.4 × 10^−7^ mol L^−1^	[88]
TP estimation	MWCNTs	Tea	Infusion	Polyphenols ox. at GCE-SWCNT/Polytyr	Amperometry	Gallic acid: 5.0 × 10^−7^ and 1.7 × 10^−4^ mol L^−1^; 8.8 × 10^−9^ mol L^−1^	[89]
Total isoflavones and evaluation of polyphenols profiles.	SPE-MWCNTs	dietary supplement/apples and pears extracts	No required (sample dilution)/LLE	Chip flow injection/separation ox at and channel SPE-MWCNTs	Chip flow injection and separation/amperometric detection	Genistein: 0.3–1.5 × 10^1^ mg L^−1^; 0.4 × 10^−1^ mg L^−1^. Phloridzin: 1.0 × 10^1^–1.5 mg 10^2^ L^−1^; 1.2 mg L^−1^	[90]
Total glycosides (TG) and total aglycones (TA) Isoflavones	SWCNTs	soy extracts	LLE	Chip separation ox at and channel Press-produced MWCNTs transducer	Chip separation/amperometric detection	Genistein and Daidzein: 2.0 × 10^−5^–5.0 × 10^−4^ mol L^−1^; 2.0 × 10^−5^ mol L^−1^	[91]
Polyphenols studies	CB	-	-	Polyphenols ox. at SPE-CB	SWV	Gallic acid: 1.0 × 10^−5^–1.0 × 10^−4^ mol L^−1^; 1.0 × 10^−6^ mol L^−1^	[92]
AOC evaluation of *o*-diphenols and *m*-phenols	CB	Olive oil	SPE	Polyphenols ox. at a Press-produced CB transducer	DPV	Tyrosol and hydroxytrosol: 1.0 × 10^−5^–7.5 × 10^−5^ mol L^−1^; 6.0 × 10^−6^ and 2.0 × 10^−5^ mol L^−1^, respectively	[93]
AOC evaluation	CB (Printex L6 carbon)-AgNPs	wine	No required (sample dilution)	Polyphenols ox. at GCE-CB/AgNPs	DPV	Gallic acid: 5.0 × 10^−7^–8.5 × 10^−6^ mol L^−1^; 6.6 × 10^−8^ mol L^−1^	[94]
AOC evaluation	GR-boron-doped	Tea	Infusion	Polyphenols ox. at GCE-GR/boron doped	DPV	Gallic acid: 1.2 × 10^−6^–1.2 × 10^−5^ mol L^−1^	[95]
AOC evaluation	GR-thermally reduced	Beer	No required (sample dilution)	Polyphenols ox. at GCE-GR/thermally reduced	DPV	Catechin: 1.2 × 10^−6^–1.2 × 10^−5^ mol L^−1^	[96]
AOC evaluation	GR reduced-Fe_2_O_3_/Chitosan	Red/white wine	No required (sample dilution)	Polyphenols ox. at GCE-GR reduced-Fe_2_O_3_/Chit	DPV	Gallic acid: 1.0 × 10^−6^ mol L^−1^–1.0 × 10^−4^ mol L^−1^ ; 1.5 × 10^−7^ mol L^−1^	[97]
AOC evaluation	GR-PEDOT-poly (styrenesulfonate)	herbs and herbal beverages	LLE/Dilution	Indirect evaluation via DPPH• ox. at SPE-GR/PEDOT/PSS	Chronoamperometry	Trolox: 5.0 × 10^−6^–3.0 × 10^−5^ mol L^−1^; 0.6 × 10^−6^ mol L^−1^	[98]
Polyphenols evaluation (proof)	Maghemite NPs-tannic acid	blueberry	LLE	Polyphenols ox. at CPE-SAMN/TA	SWV	Hydroquinone: 2.5 × 10^−5^–5.0 × 10^−4^ mol L^−1^; 8.6 × 10^−6^ mol L^−1^	[99]
AOC evaluation (*o*-diphenols)	Cerium (IV)oxide NPs	Red/white wine	No required (sample dilution)	Polyphenols ox at quinines, quinines reduction at SPE-CeO (IV)NPs	Amperometry	Gallic acid: 2.0 × 10^−6^–2.0 × 10^−5^ mol L^−1^; 1.5 × 10^−6^ mol L^−1^	[100]
Polyphenols content double index	graphitized mesoporous carbon/Chitosan	Tea	Infusion	Polyphenols ox Polyphenols ox at GCE-Chit/GMC at two potentials	FIA-amperometric(double potential)	1,2,3-THB, and (1,2,3-THB, 1,2-DHB,1,3-DHB): 0.1 × 10^−6^–1.0 × 10^−4^ mol L^−1^; 1.1 × 10^−6^ mol L^−1^ and 0.9 × 10^−6^ mol L^−1^, respectively	[101]
Polyphenols evaluation/resolve phenolic mixture	copper nanoparticles	Wine	No required (sample dilution)	epoxy–graphite-(bio-)electronic array	CV data input for artificial neural network	-	[102]

**Table 3:** Electrochemical enzyme sensors based on nanomaterial for food polyphenols evaluation: main features and strategies.

Work Aim	Nanomaterial	Enzyme/Biological Element	Immobilization	Food Matrix	Sample Pretreatment	Principle	Linear Range/LOD	Ref.
TP estimation	MWCNT-Chitosan	Laccase from Trametes versicolor	MWCNT/Chitosan electrodeposition entrapping	Sage and mint	LLE	Laccase phenols derived quinones Amperometric reduction at Gold sheet-MWCNT/CS	Rosmarinic acid: 9.1 × 10^−7^; 1.2 ×10^−5^; 2.3 × 10^−7^ mol·L^−1^	[114]
TP index	SWCNT/MWNCT	Laccases from *Trametes versicolor* (TvL) and *Trametes hirsuta* (ThL)	PAP cross-linking	Red and white wine	No required (sample dilution)	Laccase phenols derived quinones FIA-Amperometric reduction at SPE-SWCNT	Gallic Acid: 0.1–1.7 × 10^1^ mg·L^−1^; 0.1 mg·L^−1^	[115]
TP evaluation	C-MWCNT-PANI/AgNPs	laccase from *Ganoderma* sp.	Covalent onto AgNPs/C-MWCNT/PANI	Tea leaves, alcoholic beverages, pharmaceutical formulations	Infusion/no required (sample dilution)	Laccase phenols derived quinones Amperometric reduction at gold electro-C-MWCNT-PANI/AgNPs	Guaiacol: 1.0 × 10^−5^–5.0 × 10^−4^ mol·L^−1^; 5.0 × 10^−8^ mol·L^−1^	[116]
TP evaluation	C-MWCNT-PANI/NiNPs	laccase from *Ganoderma* sp.	Covalent onto NiNPs/C-MWCNT/PANI	Fruit juices	No required (sample dilution)	Laccase phenols derived quinones CV reduction at gold electrdo-C-MWCNT-PANI/NiNPs	Guaiacol: 1.0 × 10^−5^–5.0 × 10^−4^ mol·L^−1^; 5.0 × 10^−8^ mol·L^−1^	[117]
TP evaluation	GRO-PtNPs	Laccase from: *Trametes versicolor*	Nafion entrapping	tea infusions	Infusion/	Laccase phenols derived quinones Amperometric reduction at SPE-GRO/PtNPs/Nafion	Caffeic acid: 2.0 × 10^−7^–2.0 × 10^−6^ mol·L^−1^; 9.0 × 10^−8^ mol·L^−1^	[118]
TP evaluation	Fullerene-AuNPs	Laccase from: *Trametes versicolor*	Covalent onto Au-SAM/AuNPs-Linker/Fullerenols/TvL	Red and white wine	No required (sample dilution)	Laccase phenols derived quinones FIA-amperometric reduction at gold electrode-SAM/AuNPs Linker/Fullerenols	Gallic acid: 3.0 × 10^−5^–3.0 × 10^−4^ mol·L^−1^; 6.0 × 10^−6^ mol·L^−1^	[119]
TP evaluation	GRQDs-MoS_2_/nanoflakes	Laccase from: *Trametes versicolor*	Electrostatic interaction laccase/GRQDs	Red wine	No required (sample dilution)	Laccase phenols derived quinones Amperometric reduction at SPE-GRQDs-MoS_2_/nanoflakes	caffeic acid: 3.8 × 10^−7^–1.0 × 10^−4^ mol·L^−1^; 3.2 × 10^−7^ mol·L^−1^;	[120]
TP evaluation	GRO-MWCNTs	Laccase from: *Trametes versicolor*/tyrosinase from mushroom	BSA reticulated with GA/chitosan entrapping	fruit juices	Centrifugation (sample dilution)	Laccase/tyrosinase phenols derived quinones Amperometric reduction at GCE-GRO/MWCNTs	Catechol: 1.0 × 10^−6^–3.0 × 10^−4^ mol·L^−1^ ; 3.0 × 10^−7^ mol·L-^1^	[121]
TP evaluation	CB	Tyrosinase from mushroom	Entrapping	Olive oil	LLE	Laccase phenols derived quinones Amperometric reduction at Carbon paste electrode-CB	Catechol: 1.25 × 10^−8^ to 1.5 × 10^−4^ mol·L^−1^; of 6.0 × 10^−9^	[122]

## Abbreviations

ABTS•	2,2′-Azino-bis(3-ethylbenzothiazoline-6-sulphonic acid)
AOC	Antioxidant capacity
AgNPs	Silver nanoparticles
AuNPs	Gold nanoparticles
B-DD	Boron doped diamond
B-GR	Boron-doped graphene
BSA	Bis(trimethylsilyl)acetamide
CB	Carbon Black
CBNPs	Carbon black nanoparticles
CeONPs	Cerium oxide nanoparticle-based
CNTs	Carbon nanotubes
CPE	Carbon paste electrode
CS	Chitosan
CTAC	Cetyltrimethylammonium chloride
CUPRAC	Cupric ion reducing antioxidant capacity
CV	Cyclic Voltammetry
DMF	Dimetylformamide
DMSO	Dimethyl sulfoxide
DPPH•	2,2-Diphenyl-1-picrylhydrazyl
DPV	Differential pulse voltammetry
DPV	Voltammetry
FIA-AD	Flow-injection analysis with amperometric detection
FRAP	Ferric reducing ability of plasma
FTIR	Fourier transform infrared spectroscopy
GCE	Glassy carbon electrode
GEAC	Gallic acid equivalent antioxidant capacity
GR	Graphene
GNR	Graphene nanoribbon
GRO	Graphene oxide
GRQDs	Graphene quantum dots
H	High
IONPs	Iron oxide nanoparticles
L	Lower
Lacc	Laccase
LLE	Liquid–liquid extraction
LOD	Limit of detection
LOQ	Limit of quantification
LSPR	Localized surface plasmon resonance
MAE	Microwave assisted extraction
MNPs	Metal nanoparticles
*m*-phenol	Mono-phenol
MWCNTs	Multi-walled carbon nanotubes
Nanoceria	Cerium (IV) oxide NPs
N-GR	Nitrogen doped graphene
N-GR	Nitrogen-doped graphene
NM	Nanomaterials
NPs	Nanoparticles
*o*-diphenol	*Ortho*-diphenol
ORAC	Oxygen radical absorbance capacity
PANI	Polyaniline
PCs	Polyphenolic compounds
PCA	Principal component analysis
PEDOT	Poly (3,4-ethylenedioxythiophene)
PEI	Polyethylenimine
PLE	Pressurized liquid extraction
PMMA	Polymethyl metacrylate
Polytyr	Polytyrosine
PPO	Polyphenol oxidase
PSS	Poly(styrene sulfonate)
PtNPs	Platinum nanoparticles
PVA	Poly(vinyl alcohol)
QDs	Quantum dots
RhNPs	Rhodium nanoparticles
SCE	Super critical extraction
SLE	Solid-liquid extraction
SPE	Screen printed electrode
SPE	Solid phase extraction
SWCNTs	Single-walled carbon nanotubes
SWV	Square Wave Voltammetry
SWV	Square Wave Voltammetry
TA	Total aglycones
TEAC	Trolox equivalent antioxidant capacity
TEM	Transmission electron microscopy
TG	Total glycosides
T-GR	Thermally reduced graphene
TP	Total Polyphenols
TRAP	Total Reactive Antioxidant Potential
UAE	Ultrasonic assisted extraction
